# Supplementary figures and images for: The Drosophila fussel gene is required for bitter gustatory neuron differentiation acting within an Rpd3 dependent chromatin modifying complex
Source: PLoS Genet. 2019 Feb 7;15(2):e1007940. doi: 10.1371/journal.pgen.1007940 (PMC6382215; doi:10.1371/journal.pgen.1007940)

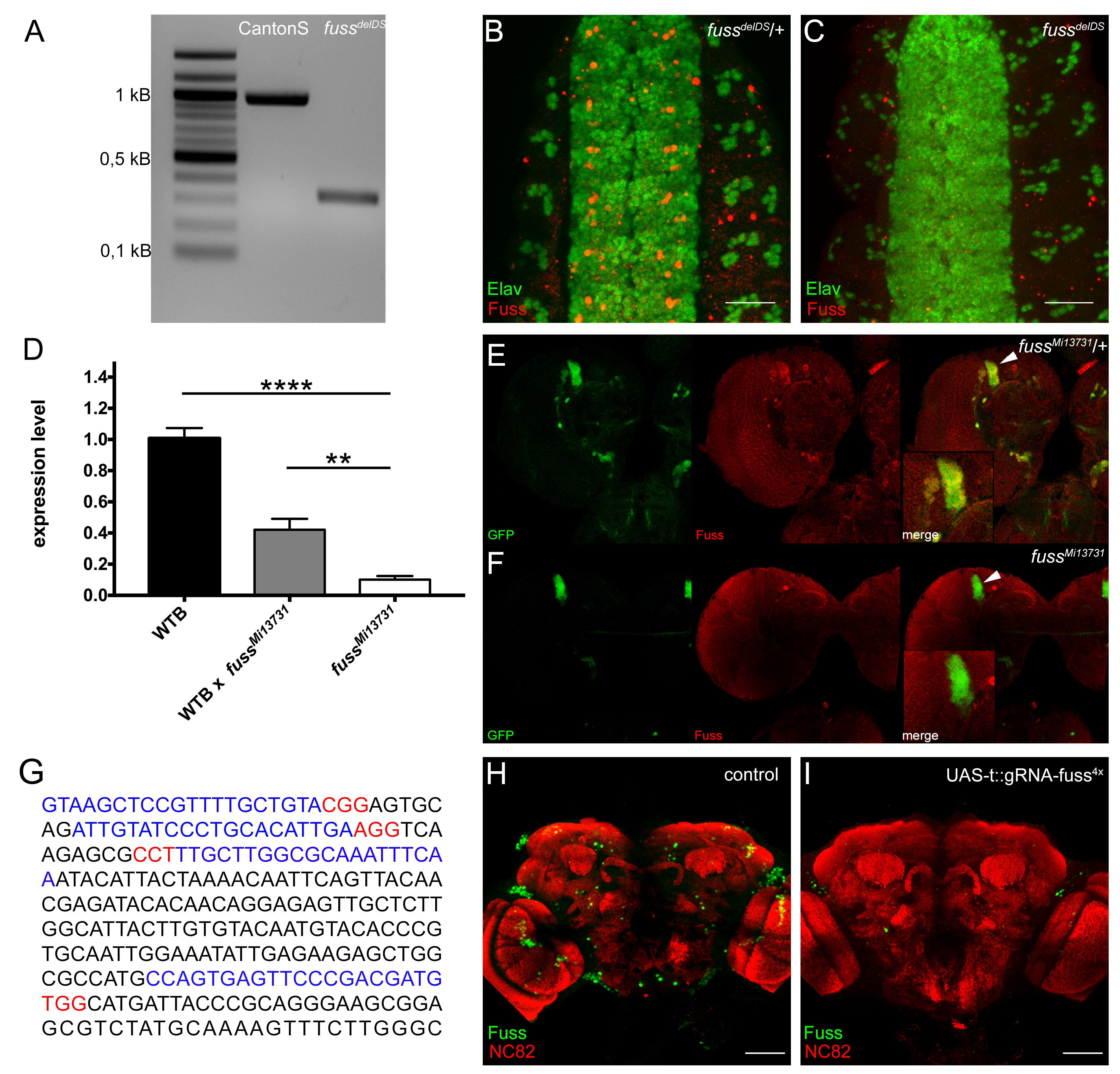

Supplement: S1 Fig — (A) Genotyping of CantonS and homozygous fussdelDS flies with fuss crispr1 seq fw and fuss crispr2 seq rv oligonucleotides via PCR of genomic DNA shows reduction of around 700bp in fussdelDS mutants as expected in contrast to control (B). Staining of heterozygous fussdelDS/+ embryos with anti-Fuss (red) and anti-Elav (green) antibodies. Scale bar indicates 25 μm. (C) Staining of homozygous fussdelDS embryos with anti-Fuss (red) and anti-Elav (green) antibodies. Scale bar indicates 25 μm. (D) Analysis of fussB and fussD transcript levels with fussBD fw and fussBD rv oligonucleotides via qPCR reveals a reduction of fussB and fussD transcript levels to 10% in homozygous fussMi13731 flies in contrast to WTB flies. n = 4 for each genotype. One-way ANOVA with post hoc Tukey´s test was used to calculate p-values. ****p<0.0001. **p<0.01. Error bars indicate SEM. (E) Anti-Fuss staining colocalizes with GFP in larval brains of heterozygous fussMi13731/+ line. (F) No anti-Fuss staining in larval brains of homozygous fussMi13731 line can be detected. Arrowhead indicates magnified cell cluster. (G) Location of the four CRISPR target sites of the UAS-t::gRNA-fuss4x construct in the DNA sequence of the Ski/Sno homology domain. (H) Adult brains of UAS-cas9/UAS-fussB-GFP; fussBD-Gal4/+ flies show normal Fuss expression pattern. Scale bar indicates 50 μm. (I) In flies of the genotype UAS-cas9/UAS-fussB-GFP; UAS-t::gRNA-fuss4x; fussBD-Gal4/+ fussB-GFP is strongly reduced. Scale bar indicates 50 μm. (TIF) [file pgen.1007940.s001.tif]

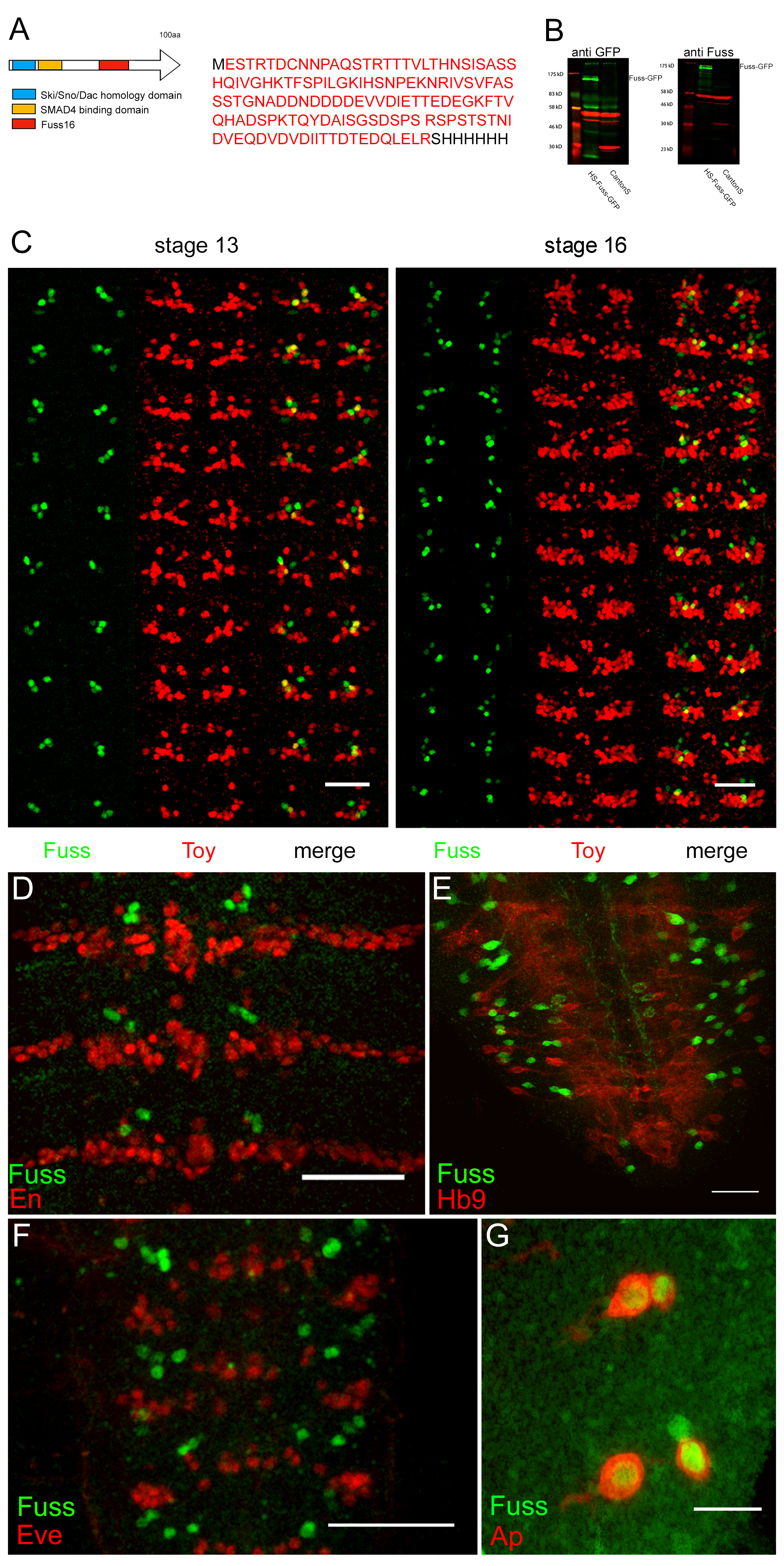

Supplement: S2 Fig — (A) Schematic representation of conserved domains and localization of the Fuss16 fragment used for immunization. Exact sequence of Fuss16-His fragment shown in red. (B) Detection of Fuss-GFP (green) from heatshock induced Fuss-GFP flies in western blots by anti-GFP and anti-Fuss antibodies. As a negative control CantonS is used and Tubulin as a housekeeper protein (red). Both antibodies recognize a predicted protein size of 112 kDa for the fusion protein. Endogenous levels of the Fuss protein cannot be detected on western blots due to the low abundance of the protein. (C) Comparison of VNC of stage 13 embryo with VNC of stage 16 embryo shows increase in number of Fuss (green) or Toy (red) cells, but only one cell per hemineuromer shows colocalization of both markers. (D) Comparison of expression pattern of interneuron marker Engrailed (red) and Fuss (green) visualized by antibody staining in embryonic VNC. (E) Fuss expression pattern as revealed by expression of GFP (green) by heterozygous fussMi13731/+ in larval brains does not colocalize with LacZ (red) driven by Hb9-GAL4 line. (F) Even skipped (red), a motor neuron marker, is not expressed in Fuss neurons (green) visualized by antibody staining in embryonic VNC. (G) Ventral Apterous cells marked by expression of CD8-RFP (red) with ap-Gal4 are positive for Fuss (green) expression in larval VNC. Scale bars indicate 30 μm (C, D, E, F) and 10 μm (G). (TIF) [file pgen.1007940.s002.tif]

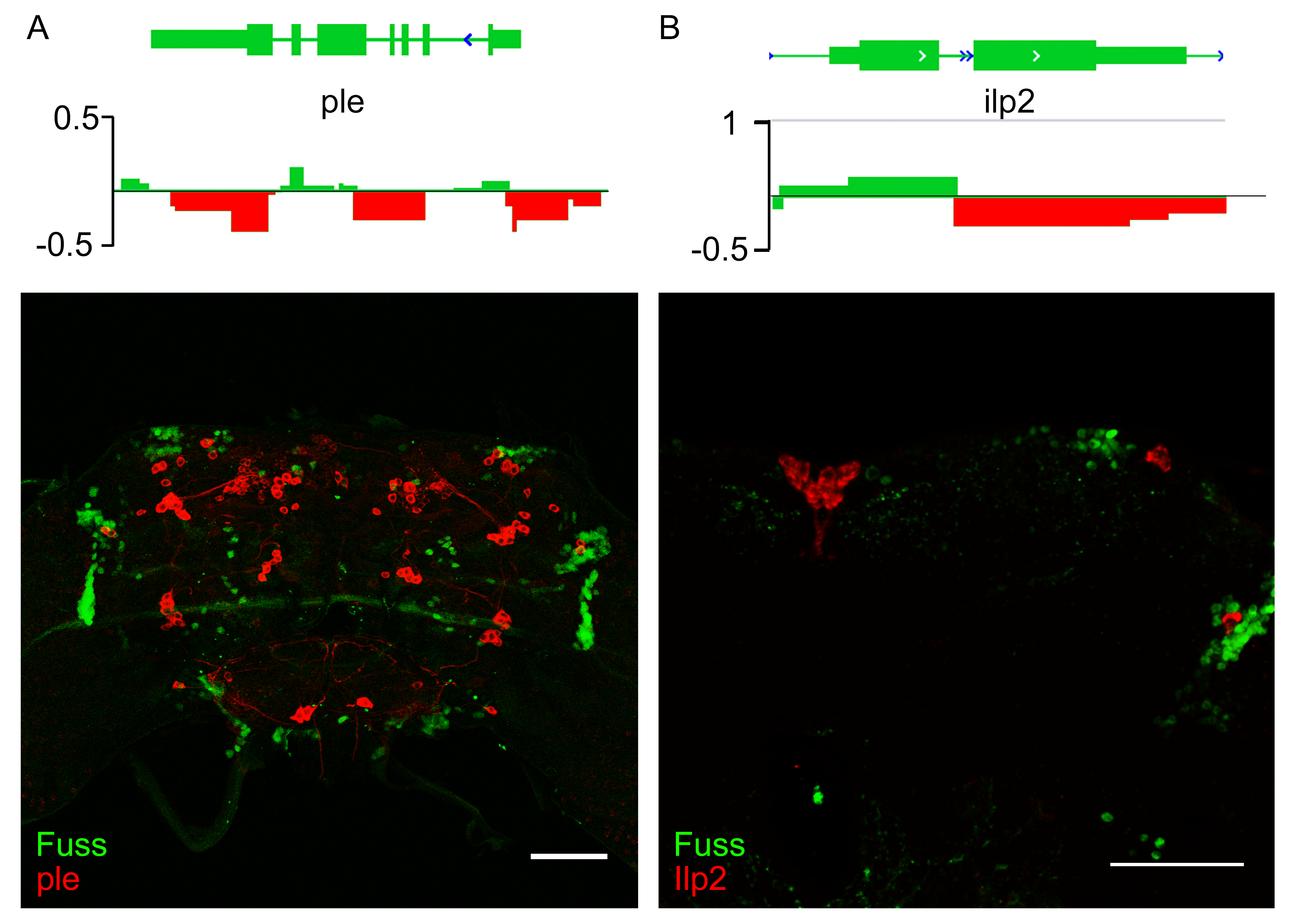

Supplement: S3 Fig — (A) pale (ple) is weakly bound by Dam-PolII as revealed by TaDa and no colocalization is observed between Ple positive cells (red) and GFP expressed by the heterozygous fussMi13731/+ reporter line (green) in whole adult brains. Overlap between signals arises from different optical slices and not from colocalization. (B) insulin like peptide 2 (ilp2) is weakly bound by Dam-PolII as revealed by TaDa. Confocal slices covering the pars intercerebralis and a part of the adult brain hemisphere show no colocalization between insulin producing cells labeled with anti-Ilp2 antibody (red) and Fuss neurons labeled with anti-Fuss antibody (green). In (A) and (B) regions bound stronger by Dam-PolII than by Dam are depicted in green, whereas regions bound stronger by Dam than by Dam-PolII are depicted in red. Scale bars indicate 50 μm. (TIF) [file pgen.1007940.s003.tif]

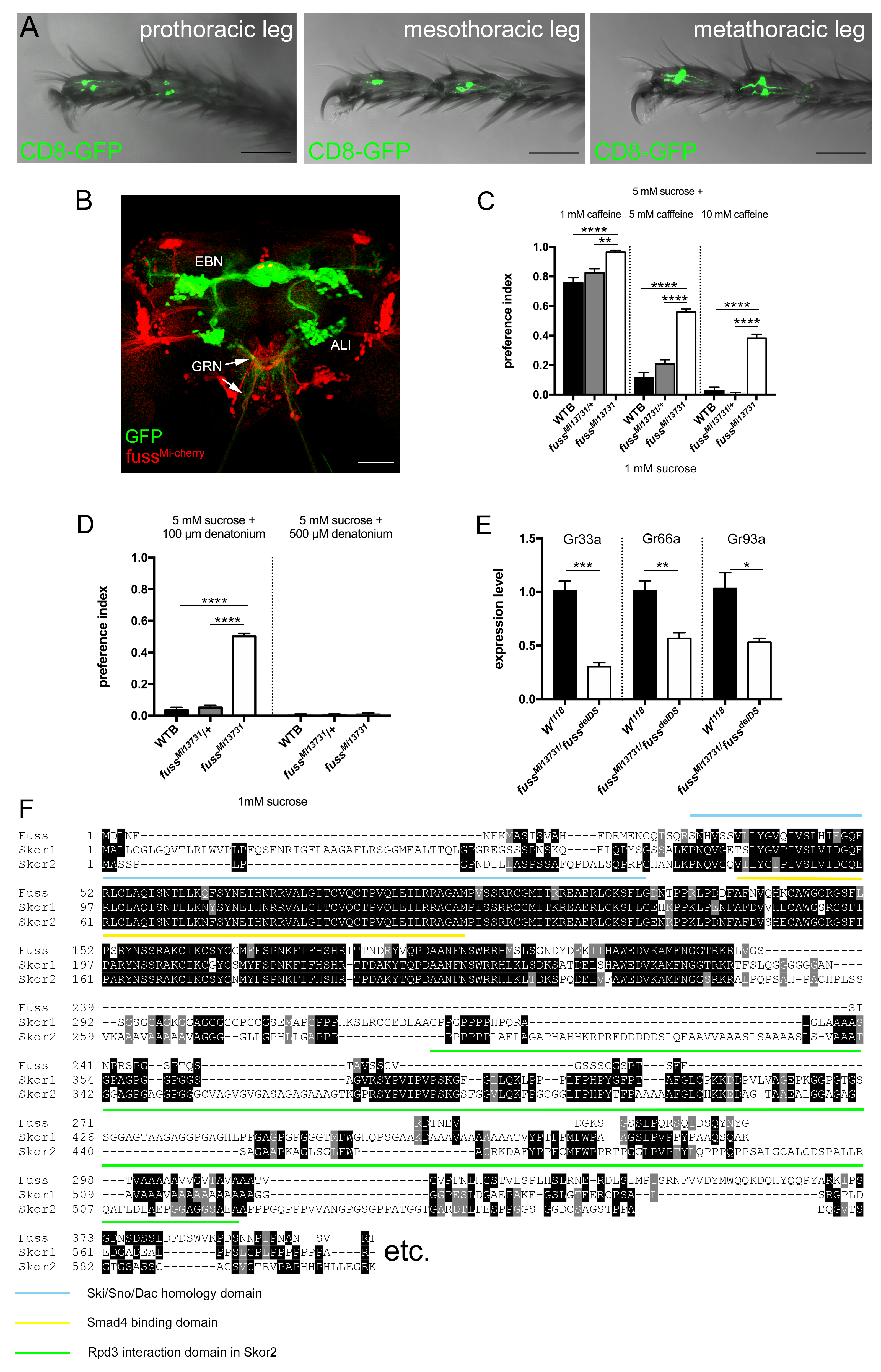

Supplement: S4 Fig — (A) Expression of UAS-CD8-GFP with fussBD-Gal4 reveals four GRNs located in the two last tarsal segments of the prothoracic, mesothoracic and metatoracic leg. Scale bars indicate 50 μm. (B) GFP expression from Poxn-Gal4-13-1 is not overlapping with Cherry expression from fussMi-cherry reporter line in neurons of the adult CNS. Overlap can only be observed in GRN nerve fibers from proboscis. EBN = ellipsoid body neurons. ALI = Antennal lobe interneurons. Scale bar indicates 50 μm. (C) Homozygous fussMi13731 flies show reduced caffeine sensation also at lower concentrations compared to heterozygous fussMi13731 x WTB and WTB flies. n = 4–9 for each genotype. One-way ANOVA with post hoc Tukey´s test was used to calculate p-values. **p<0.01 ****p<0.0001. Error bars indicate SEM. (D) Homozygous fussMi13731 mutant flies show reduced sensation of denatonium benzoate compared to heterozygous fussMi13731 x WTB and WTB flies at a concentration of 100 μm. At 500 μm denatonium benzoate effect of homozygous fussMi13731 flies is reversed to control levels. n = 4–5 for each genotype. One-way ANOVA with post hoc Tukey´s test was used to calculate p-values. ****p<0.0001. Error bars indicate SEM. (E) Transheterozygous fussMi13731/fussdelDS mutants show reduced transcript levels for Gr33a, Gr66a and Gr93a in contrast to W1118 control. n = 4 for each genotype. One-way ANOVA with post hoc Tukey´s test was used to calculate p-values. ***p<0.001. **p<0.01. *p<0.05. Error bars indicate SEM. (F) Alignment of Drosophila Fuss with mouse Skor1 and Skor2. Ski/Sno/Dac homology domain, SMAD4 binding domain and proposed Rpd3 interaction fragment in Skor2 are displayed by colored lines as described. (TIF) [file pgen.1007940.s004.tif]
